# Characterizing roots and water uptake in a ground cover rice production system

**DOI:** 10.1371/journal.pone.0180713

**Published:** 2017-07-07

**Authors:** Sen Li, Qiang Zuo, Xiaoyu Wang, Wenwen Ma, Xinxin Jin, Jianchu Shi, Alon Ben-Gal

**Affiliations:** 1 Department of Soil and Water Sciences, China Agricultural University, Beijing, China; 2 Key Laboratory of Plant-Soil Interactions, Ministry of Education, Beijing, China; 3 Key Laboratory of Arable Land Conservation (North China), Ministry of Agriculture, Beijing, China; 4 Soil, Water and Environmental Sciences, Agricultural Research Organization, Gilat Research Center, Negev, Israel; Duzce Universitesi, TURKEY

## Abstract

**Background and aims:**

Water-saving ground cover rice production systems (GCRPS) are gaining popularity in many parts of the world. We aimed to describe the characteristics of root growth, morphology, distribution, and water uptake for a GCRPS.

**Methods:**

A traditional paddy rice production system (TPRPS) was compared with GCRPS in greenhouse and field experiments. In the greenhouse, GCRPS where root zone average soil water content was kept near saturation (GCRPS_sat_), field capacity (GCRPS_fwc_) and 80% field capacity (GCRPS_80%_), were evaluated. In a two-year field experiment, GCRPS_sat_ and GCRPS_80%_ were applied.

**Results:**

Similar results were found in greenhouse and field experiments. Before mid-tillering the upper soil temperature was higher for GCRPS, leading to enhanced root dry weight, length, surface area, specific root length, and smaller diameter of roots but lower water uptake rate per root length compared to TPRPS. In subsequent growth stages, the reduced soil water content under GCRPS caused that the preponderance of root growth under GCRPS_sat_ disappeared in comparison to TPRPS. Under other GCRPS treatments (GCRPS_fwc_ and GCRPS_80%_), significant limitation on root growth, bigger root diameter and higher water uptake rate per root length were found.

**Conclusions:**

Discrepancies in soil water and temperature between TPRPS and GCRPS caused adjustments to root growth, morphology, distribution and function. Even though drought stress was inevitable after mid-tillering under GCRPS, especially GCRPS_80%_, similar or even enhanced root water uptake capacity in comparison to TPRPS might promote allocation of photosynthetic products to shoots and increase water productivity.

## Introduction

Rice (*Oryza sativa* L.) is one of the most important grain crops for more than 50% of the world’s population, accounting for approximately 20% of total energy intake, and an annual increase of 8–10 million tons is estimated necessary to meet future needs [1]. China is the largest rice producer and consumer in the world, and the area under rice accounts for about 30% of the country’s total farmland while consuming approximately 70% of water resources directed to agriculture [2]. Cultivation in a traditional paddy rice production system (TPRPS) is typically characterized by luxurious water consumption and low efficiency [3]. Rapidly increasing population and global water shortage make development of water-saving rice production technologies inevitable, especially in China [4, 5].

Among available rice production technologies, the ground cover rice production system (GCRPS) is increasingly adopted in countries including China because of its contribution to both saving water and increasing yields [6–9]. Initially proposed in cool mountainous areas in 1980s [10], GCRPS has been successfully extended to more than 4 million hectares in China so far [11, 12]. In a GCRPS, rice is planted in the strip soil beds, on which mulch such as plastic film or crop straw is placed instead of standing water [13]. The most significant aspects discriminating TPRPS from GCRPS lie in the transformation of root zone soil water status from completely saturated and anaerobic to partially unsaturated and aerobic and increased temperature of the upper soil layer especially during the early growth season. These alterations in soil water and temperature are expected to affect root growth, morphology, distribution, and subsequently water uptake [14–16]. Furthermore, plant growth and yield might also be impacted since the root system is not only the main organ for water and nutrient absorption but also crucial to synthesis of hormones, organic acids, and amino acids [17, 18]. Study of the characteristics of root growth, morphology, distribution, and water uptake in a GCRPS is expected to advance understanding of its water-saving and yield-increasing mechanisms.

In recent years, with the rapid expansion of GCRPS, efforts have been made to investigate this novel system regarding water consumption, shoot growth and yield formation. The main results indicate that, relative to TPRPS, water consumption and transpiration in a GCRPS were significantly decreased, while plant growth and grain yields were promoted [13, 19–21]. However, there are few studies investigating root systems or root water uptake in a GCRPS probably because of the difficult and time-consuming features of obtaining root information in the field. The main results regarding roots under GCRPS can be summarized as: (1) roots observed under GCRPS were predominately white while in TPRPS they were brown or black [22]; (2) compared with TPRPS, root growth under GCRPS was significantly stimulated during early and middle growth stages while repressed during late growth stages [23]; (3) the total root length and root surface area in a GCRPS were always lower than those in a TPRPS at both heading and maturity stages, and the corresponding specific root length (the length per root dry weight) was also smaller due to a larger root diameter [24]. These fragmentary preliminary findings do not allow understanding root growth and function, and are insufficient for further improvement and extension of GCRPS. Using TPRPS as a control, a two-year field experiment and a greenhouse experiment were conducted to comprehensively analyze the dynamic characteristics of root growth, morphology, distribution, and water uptake in a GCRPS.

## Materials and methods

### Field experiment (Exp. 1)

A field experiment, described in detail by Jin et al. [19], was conducted from April to September in 2013 and 2014 at Fangxian Agricultural Bureau farm in Shiyan, Hubei province, China. Nine independent plots (9 m wide by 10 m long) were prepared before rice transplanting, and each plot was puddled, leveled, and separated into five strip soil beds (1.56 m in width and 9.4 m in length) surrounded by 0.15 m wide and 0.15 m deep furrows. After the application of basal fertilizers, the soil beds in 6 random plots were covered with 5 μm thick and 1.7 m wide plastic film. On April 28, 2013 and April 29, 2014, two identical rice seedlings (*Oryza sativa* L. *cv*. *Yixiang* 3728) were transplanted per hill into the soil beds at 26 cm row and 18 cm plant spacing. Rice was harvested on September 10, 2013 (135 days after transplanting, DAT) and September 19, 2014 (143 DAT).

In the three plots without plastic film for treatment TPRPS, irrigation was applied to maintain an average water depth on the soil beds between 2 and 5 cm. Three plots with plastic film were irrigated continuously through the furrows to keep the average water depth in the furrows between 10 and 15 cm, without standing water on the soil beds themselves. Since the measured root zone (0–40 cm soil layer) average water content was close to saturation, this treatment was named GCRPS_sat_. The remaining three plots with plastic film, called GCRPS_80%_, were managed identically to GCRPS_sat_ before mid-tillering stage, and then transient irrigation was intermittently implemented by filling the furrows to maintain root zone average soil water content between 80% and 100% of field water capacity.

Soil water content under GCRPS was measured every 2 days with a capacitance probe (Diviner 2000, Sentek, Australia) at 10 cm intervals from soil surface to 60 cm depth. For each treatment, soil temperature at the depths of 5 (with 3 replications), 10 and 20 cm (only one replication) was measured hourly by multipoint temperature instruments (CB-0221, Yaxin, China). After 85 DAT in 2014, some failure of the temperature probes at 10 and 20 cm occurred, resulting in incomplete data. Plants were sampled during the mid-tillering, max-tillering, panicle initiation, anthesis, and maturity stages (34, 53, 78, 99 and 135 DAT in 2013; 34, 51, 78, 99 and 143 DAT in 2014). At each sampling event, 8 hills of rice (0.4 m^2^) were removed from each plot. Above ground biomass was determined after oven-drying at 70°C to constant weight. The roots of three hills in each plot for the last sampling in 2013 and one hill in each plot for each sampling in 2014 were excavated with a steel tube (50 cm in length and 15 cm in diameter). Soil and roots were sampled at 5 cm intervals from soil surface to 20 cm depth and 10 cm intervals downwards. The roots in each soil layer were washed on a 0.5 mm diameter sieve, scanned (SNAPSCAN 1236, AGFA, Germany) and analyzed with the WinRHIZO software package (Regent Instruments Inc., Canada) for root length, diameter and surface area. Generally, root diameter of rice is less than 2 mm, and roots with 0.3–2 mm diameter are considered adventitious while smaller roots are considered laterals [25]. Lateral roots with 0.15–0.3 mm diameter can branch into finer roots [26]. The roots were therefore divided into three groups according to their diameters (0–0.15, 0.15–0.3, and 0.3–2 mm) in this study. Root dry weight was determined by oven-drying to constant weight at 70°C. According to the measured length and dry weight of roots and the corresponding soil volume, specific root length and root length density were calculated [27]. The maximal rooting depth under the three treatments throughout the growing season was nearly identical and equal to 40 cm. Thus the root zone for each treatment was confined to 0–40 cm.

To compare root distributions under various treatments, root length density was normalized as follows [28]:

$$L_{nrd}\left(z_{r}\right)=\frac{L_{d}\left(z_{r}\right)}{\int_{0}^{1}L_{d}\left(z_{r}\right)dz_{r}}$$ (1)

where *z*_*r*_ (= *z*/*L*_*r*_) is the normalized depth ranging from 0 to 1, and *z* is the vertical coordinate originating from soil surface and positive downward (cm); *L*_*r*_ is the rooting depth (cm); *L*_*d*_(*z*_*r*_) is the root length density (cm cm^-3^); *L*_*nrd*_(*z*_*r*_) is the normalized root length density. All the normalized root length density from different growth stages, treatments and planting years were pooled together and fitted as a general function [29]:

$$L_{nrd}\left(z_{r}\right)=a{\left(1-z_{r}\right)}^{a-1}$$ (2)

where *a* is the fitted coefficient, representing the normalized root length density at soil surface.

The actual water uptake rate per root length (root water uptake coefficient, *c*_*ra*_, cm^3^ cm^-1^ d^-1^) under each treatment was estimated as follows [30, 31]:

$$c_{ra}=\frac{T_{a}}{R_{L}}$$ (3)

where *T*_*a*_ is the actual transpiration rate (cm^3^ cm^-2^ d^-1^), estimated through water balance method according to Jin et al. [19], and *R*_*L*_ is the total root length per unit soil surface area (cm cm^-2^). To compare the root water uptake capacity under various treatment conditions, the actual root water uptake coefficient was divided by a dimensionless reduction function to exclude the effect of water stress [30, 32]:

$$c_{rp}=\frac{c_{ra}}{\gamma (h)}$$ (4)

where *c*_*rp*_ is the potential root water uptake coefficient (cm^3^ cm^-1^ d^-1^); *h* is the soil matric potential (cm), transformed with the measured soil water content according to the soil water retention curve [19]; *γ*(*h*) is the dimensionless reduction function corresponding to water stress, delineated either as linear [30, 33] or non-linear [34, 35]. For simplification, a piecewise linear function [30] was chosen in this study:

$$\gamma (h)=\left\{ \begin{array}{ll} 1 & \;h_{L}<h\leq 0\\ \frac{h-h_{W}}{h_{L}-h_{W}} & \;h_{W}<h\leq h_{L} \end{array}\right.$$ (5)

where *h*_*L*_ and *h*_*W*_ are threshold values for root water uptake, chosen respectively as -250 and -15000 cm for rice [36–39].

### Greenhouse experiment (Exp. 2)

A greenhouse experiment, described in detail by Jin et al. [19], was conducted to confirm the effects of GCRPS on root characteristics found in Exp. 1. Forty-eight soil columns, 50 cm in depth and 15 cm in diameter, were prepared. A single germinated rice (same cultivar as in Exp. 1) seed was directly transplanted into each column on May 8, 2013, and grown until August 15 (99 DAT). Plants were uniformly cultured with a thin water layer over the soil surface until 22 DAT, when four specific irrigation treatments were initialized. The three treatments of TPRPS, GCRPS_sat_ and GCRPS_80%_ were managed as described in Exp. 1. The remaining treatment was GCRPS_fwc_, under which the root zone average soil water content was maintained from 100% to 120% of field water capacity.

Soil temperature at the depth of 5, 10, and 20 cm from the soil surface under each treatment was monitored as described for Exp. 1. Three columns under each treatment were randomly chosen and weighed daily at 18:00, and daily evapotranspiration (mm) was determined according to water loss and soil surface area. Under TPRPS, daily water surface evaporation (mm) was measured by a pan (15 cm in diameter) located under canopies. A total of 4 sampling events with three replications were carried out on 39, 59, 79 and 99 DAT, corresponding to the main growth stages of early tillering, mid-tillering, max-tillering, and panicle initiation, respectively. Above ground biomass was determined after oven-drying at 70°C to constant weight as in Exp. 1. Columns were opened to sample soil starting from surface to rooting depth at 5 cm intervals. Sampled soil was dried to constant weight at 105°C to determine gravimetric water content, which was subsequently transformed to volumetric water content according to soil bulk density [19]. Roots in each soil layer were washed, scanned, analyzed and dried as described for Exp. 1.

### Statistical analysis

Analysis of variance was performed with the general linear model procedure of statistical software package SPSS 20.0 (International Business Machines Corporation, USA). The statistical model included sources of variation due to year, treatment, and their interaction. Statistical significance was evaluated on the least significant difference at 0.05 probability level. No significant differences were found between the comparable stages for the two years regarding root dry weight, length, diameter, root length density, or actual and potential root water uptake coefficients (Table 1). Consequently, only the root data in 2014 are presented and further analyzed in this study.

**Table 1:** Analysis of variance (ANOVA) *F*-statistics to assess the effects of planting year (2013 and 2014) and water treatment (TPRPS, traditional paddy rice production system; GCRPS_sat_ and GCRPS_80%_, ground cover rice production system keeping root zone average soil water content near saturation and 80–100% of field water capacity, respectively) on root characteristics of rice at maturity in Exp. 1.

Source	Degree of freedom	ANOVA, *F* value										
		Root length density in different soil layers						Total root length	Total root dry weight	Average root diameter	Actual root water uptake coefficient	Potential root water uptake coefficient
		0–5 cm	5–10 cm	10–15 cm	15–20 cm	20–30 cm	30–40 cm					
Treatment	2	53.27***	28.25***	26.51***	2.97 ns	0.20 ns	1.00 ns	23.43***	11.12***	23.58***	32.24***	60.59***
Year	1	0.07 ns	1.18 ns	2.55 ns	2.13 ns	0.04 ns	3.23 ns	0.69 ns	1.65 ns	0.17 ns	2.53 ns	3.49 ns
Year×Treatment	2	0.30 ns	0.46 ns	0.83 ns	2.04 ns	0.40 ns	1.67 ns	0.19 ns	0.39 ns	0.44 ns	0.25 ns	0.41 ns

*** significant at 0.001 probability level; ns: not significant.

## Results

### Soil water and temperature conditions

**Fig 1:**
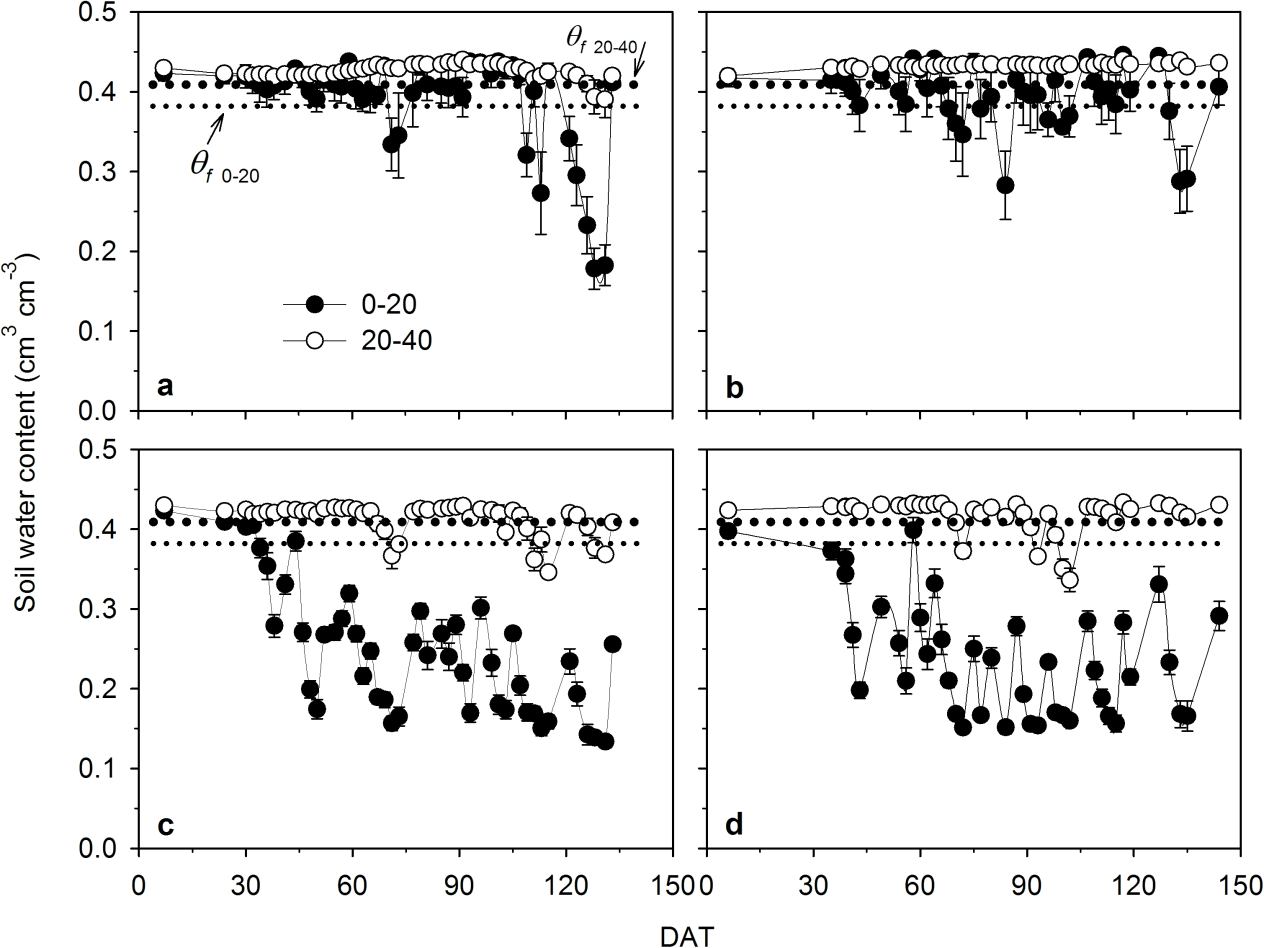
**Dynamics of averaged soil water content at different soil layers under GCRPS_sat_ and GCRPS_80%_ in 2013 (a and c) and 2014 (b and d) for Exp. 1.** GCRPS_sat_ and GCRPS_80%_, ground cover rice production system keeping root zone average soil water content near saturation and 80–100% of field water capacity, respectively. Vertical error bars represent standard errors (with the replicate number of 3 for GCRPS_sat_ and 9 for GCRPS_80%_, respectively), and DAT is days after transplanting. The bold and fine dotted lines indicate the field water capacity of 0–20 (*θ*_*f* 0–20_) and 20–40 cm (*θ*_*f* 20–40_) soil layers, respectively.

In Exps. 1 and 2, the root zone under TPRPS treatment was entirely saturated as design except during the traditional soil drying periods in Exp. 1 (66–72 and 113–135 DAT in 2013; 78–85 and 121–143 DAT in 2014). In Exp. 1, the root zone average soil water content except during the soil drying periods was around 0.41 cm^3^ cm^-3^ under GCRPS_sat_ (Fig 1A and 1B), corresponding to 91% of the average saturated water content of the two soil layers (0.45 cm^3^ cm^-3^). Under GCRPS_80%_, it was around 0.33 cm^3^ cm^-3^ (Fig 1C and 1D), corresponding to 83% of the average field water capacity of the two soil layers (0.40 cm^3^ cm^-3^). In Exp. 2, average soil water content of about 0.45 cm^3^ cm^-3^ was consistently maintained under GCRPS_sat_, corresponding to 88% of the saturated water content (0.51 cm^3^ cm^-3^). Under GCRPS_fwc_ and GCRPS_80%_, starting at saturation, soil water content declined slowly until 58 DAT, and then was maintained at around an average of 0.26 cm^3^ cm^-3^ (108% of the field water capacity) and 0.22 cm^3^ cm^-3^ (89% of the field water capacity), respectively (Fig 2B in Jin et al. [19]).

**Fig 2:**
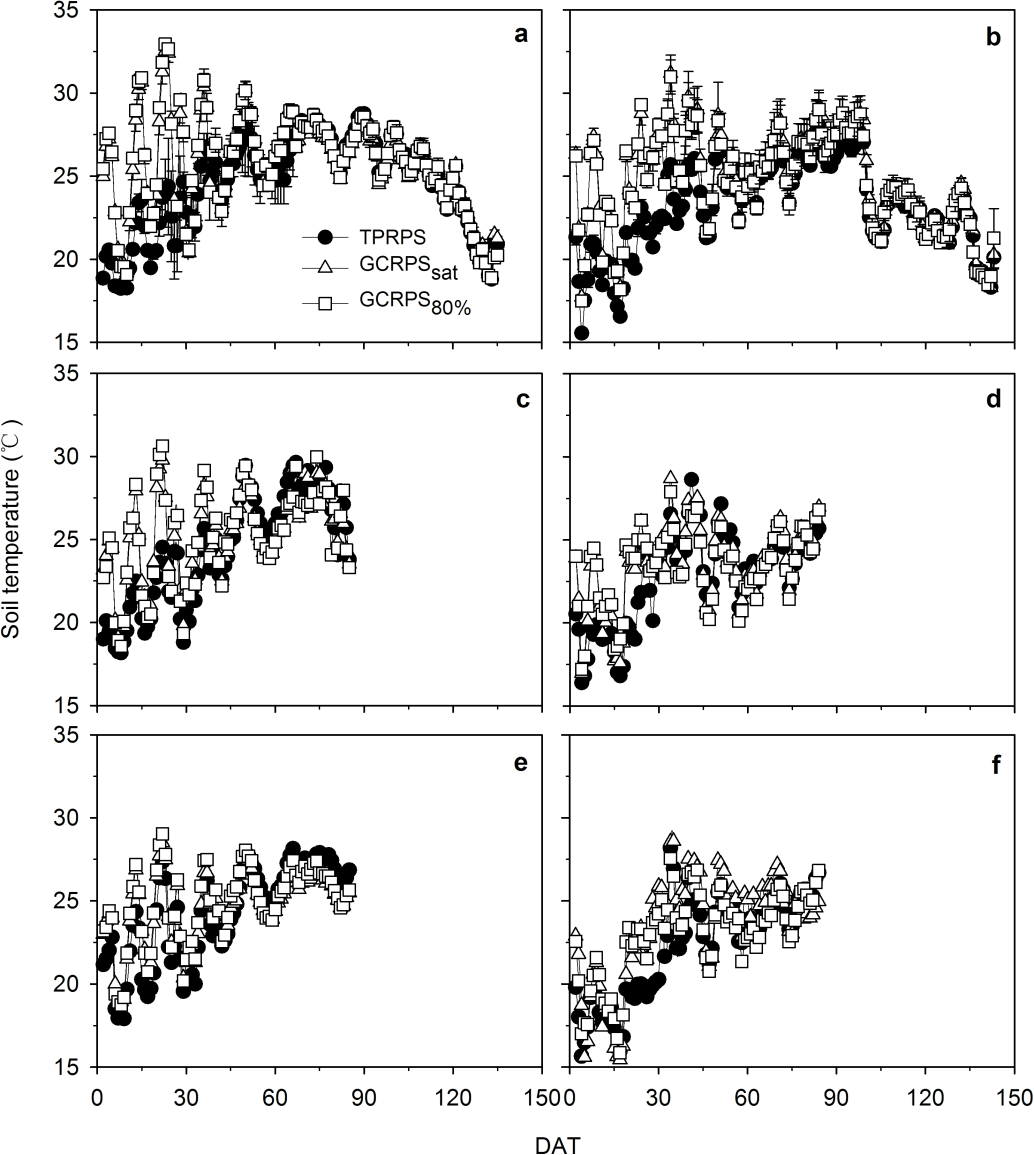
**Dynamics of daily soil temperature at depth of 5, 10 and 20 cm in 2013 (a, c and e) and 2014 (b, d and f) for Exp. 1.** TPRPS, traditional paddy rice production system; GCRPS_sat_ and GCRPS_80%_, ground cover rice production system keeping root zone average soil water content near saturation and 80–100% of field water capacity, respectively. Vertical error bars for 5 cm depth represent standard errors of 3 replications, and DAT is days after transplanting.

During the full growing seasons of rice in Exp. 1, there was no significant difference in the soil temperature at 5 cm depth between GCRPS_sat_ and GCRPS_80%_ (*p* > 0.05), but both were about 20% significantly higher (*p* < 0.001) than that under TPRPS before mid-tillering (34 DAT) (Fig 2). The warming effect under GCRPS before mid-tillering was also evident deeper in the soil, but weakened as a function of depth with temperatures around 13% higher at 10 cm and 8% higher at 20 cm depth. During the entire experimental period in Exp. 2, the difference of soil temperature between GCRPS_sat_, GCRPS_fwc_ and GCRPS_80%_ was not significant, and the warming effect under GCRPS compared to TPRPS was also found as more than 5% at the three depths (5, 10 and 20 cm) and weakened with plant growth.

### Rice growth and root characteristics

Compared to TPRPS in both Exps. 1 and 2, transpiration consumption was limited under GCRPS, while rice growth was significantly promoted with higher leaf area index, above ground biomass, and grain yield, except under GCRPS_80%_ in Exp. 2 due to serious water stress [19].

**Fig 3:**
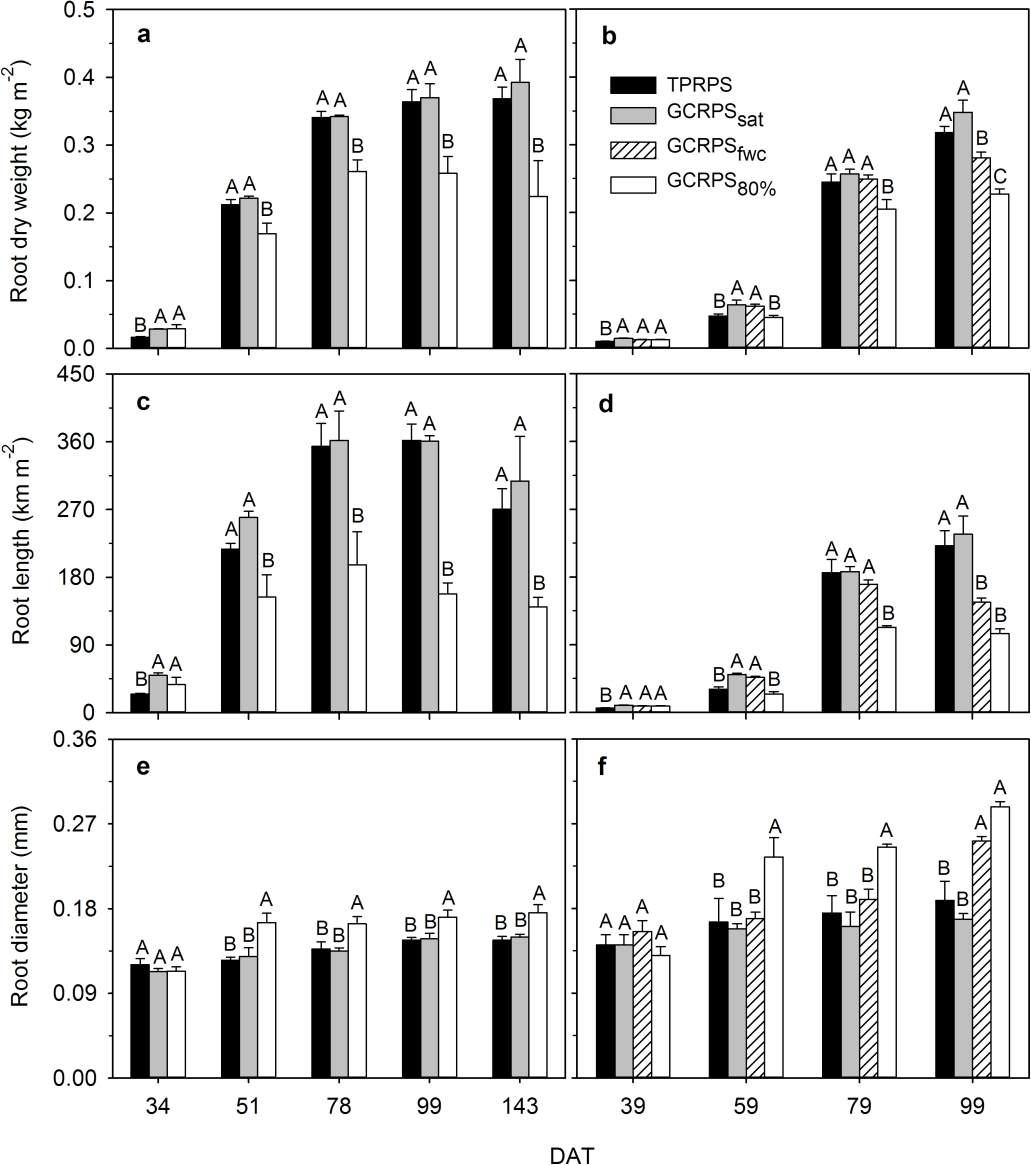
**Dynamics of root dry weight, length and diameter for rice in Exp. 1 in 2014 (a, c and e) and Exp. 2 (b, d and f).** TPRPS, traditional paddy rice production system; GCRPS_sat_, GCRPS_fwc_ and GCRPS_80%_, ground cover rice production system keeping root zone average soil water content near saturation, 100–120% and 80–100% of field water capacity, respectively. Different capital letters between treatments indicate significant difference at 0.05 probability level on given dates. Vertical error bars represent standard errors of 3 replications, and DAT is days after transplanting.

**Fig 4:**
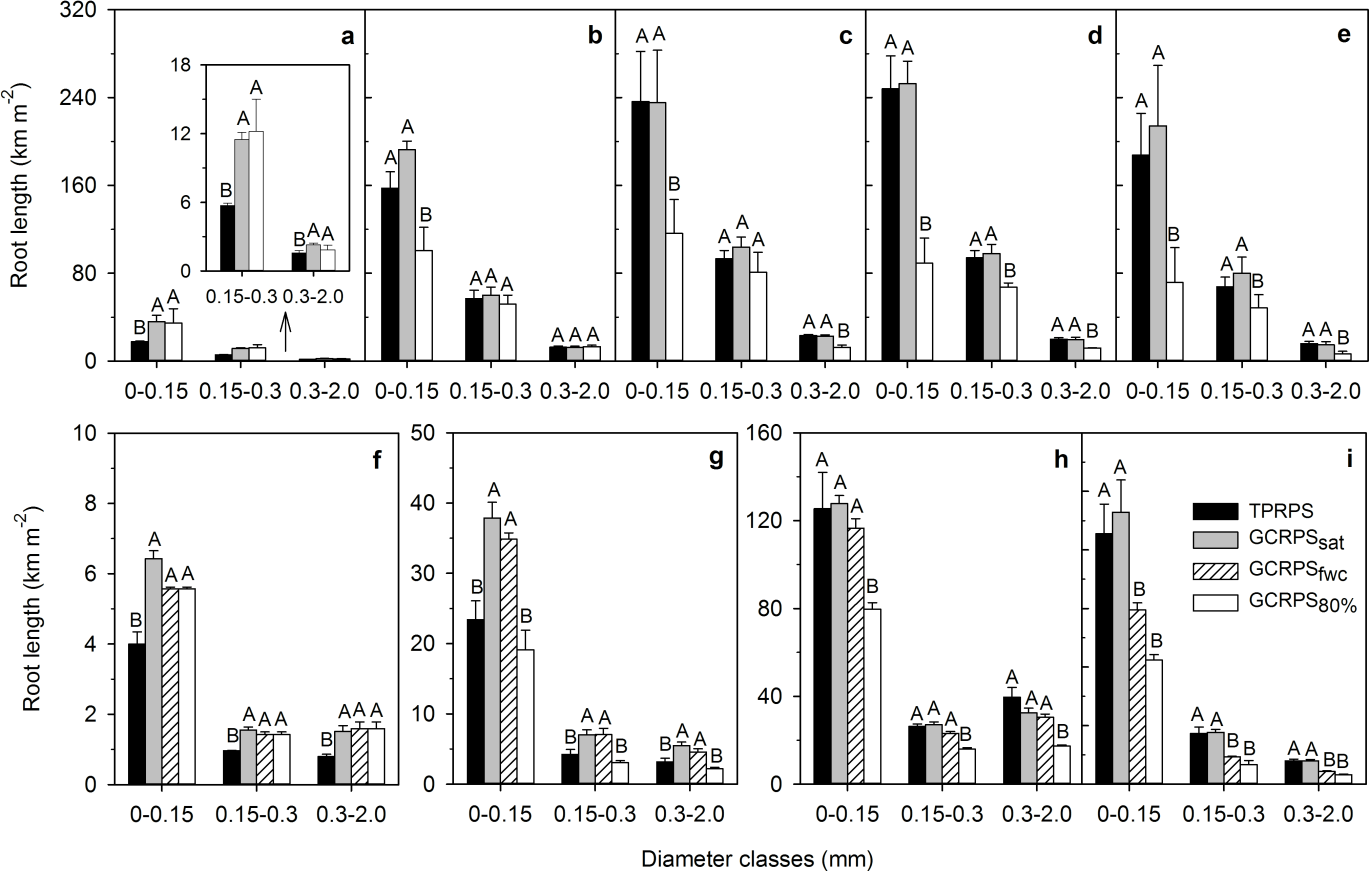
**Root length as a function of root diameter and treatment on (a) 34 DAT, (b) 51 DAT, (c) 78 DAT, (d) 99 DAT and (e) 143 DAT in Exp. 1 in 2014 and (f) 39 DAT, (g) 59 DAT, (h) 79 DAT and (i) 99 DAT in Exp. 2.** TPRPS, traditional paddy rice production system; GCRPS_sat_, GCRPS_fwc_ and GCRPS_80%_, ground cover rice production system keeping root zone average soil water content near saturation,100–120% and 80–100% of field water capacity, respectively. Different capital letters between treatments indicate significant difference at 0.05 probability level for each diameter class at each date. Vertical error bars represent standard errors of 3 replications, and DAT is days after transplanting.

Under each treatment in Exps. 1 and 2, root growth was very vigorous before panicle initiation and then slowed down especially under GCRPS_80%_ (Fig 3A–3D). During the early growth period (before mid-tillering), compared to TPRPS, root system under GCRPS grew more vigorously with higher dry weight and length (Fig 3A–3D), which was supported by roots in various diameter classes (Fig 4A and 4F). No significant difference, however, was found in average root diameter between treatments (Fig 3E and 3F). Afterwards, roots under TPRPS grew more quickly, maintaining dry weight and length similar to that under GCRPS_sat_ and significantly higher than GCRPS_fwc_ and GCRPS_80%_. No significant difference continued to be found between TPRPS and GCRPS_sat_ in diameter of roots, but under GCRPS_80%_ and GCRPS_fwc_ it was significantly larger. Root length under GCRPS_80%_ first significantly decreased compared to other GCRPS treatments, especially for the roots with 0–0.15 mm diameter. Finally, root length of various diameter classes under GCRPS_fwc_ and GCRPS_80%_ was significantly less than that under TPRPS and GCRPS_sat_ (Fig 4B–4E and 4G–4I).

**Fig 5:**
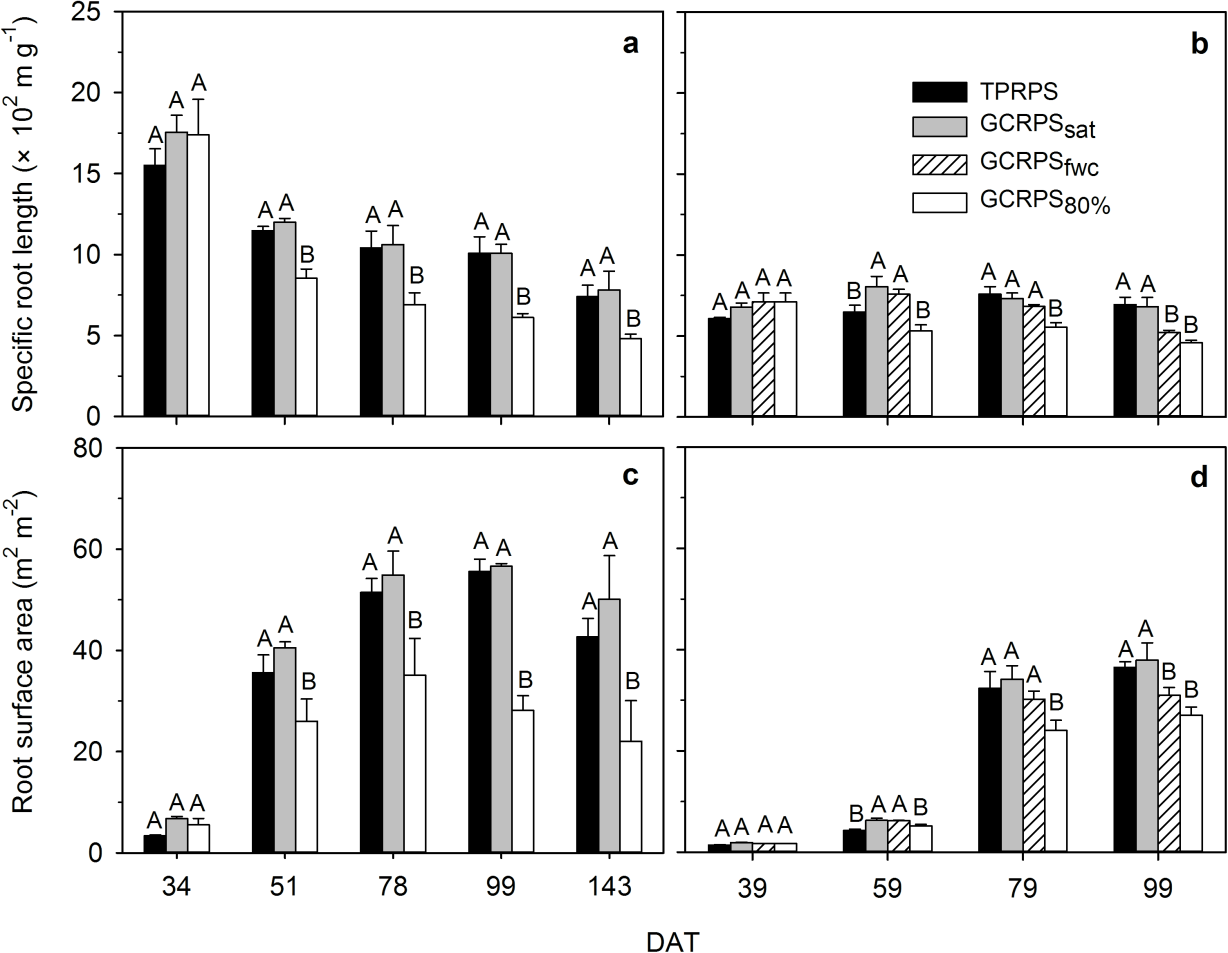
**Dynamics of specific root length and root surface area for rice in Exp. 1 in 2014 (a and c) and Exp. 2 (b and d).** TPRPS, traditional paddy rice production system; GCRPS_sat_, GCRPS_fwc_ and GCRPS_80%_, ground cover rice production system keeping root zone average soil water content near saturation,100–120% and 80–100% of field water capacity, respectively. Different capital letters between treatments indicate significant difference at 0.05 probability level on given dates. Vertical error bars represent standard errors of 3 replications, and DAT is days after transplanting.

During the early growth period, no significant difference was found in specific root length and root surface area between TPRPS and GCRPS in the two experiments (Fig 5). Afterwards, the specific root length and root surface area for TPRPS and GCRPS_sat_ were similar and significantly greater than those under GCRPS_80%_ in Exp. 1 (Fig 5A and 5C). The specific root length and root surface area under GCRPS_80%_ initially decreased in Exp. 2 compared to other GCRPS treatments, eventually those under GCRPS_fwc_ and GCRPS_80%_ were significantly smaller than that under TPRPS and GCRPS_sat_ at 99 DAT (Fig 5B and 5D).

**Fig 6:**
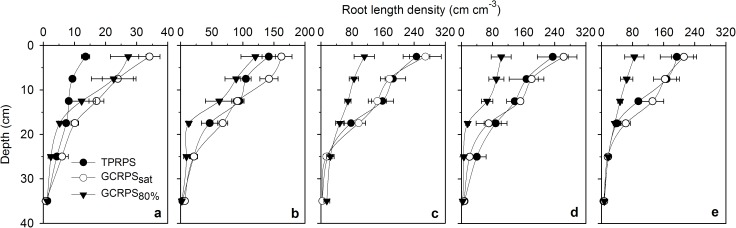
**Distributions of root length density for rice on (a) 34 DAT, (b) 51 DAT, (c) 78 DAT, (d) 99 DAT and (e) 143 DAT in Exp. 1 in 2014.** TPRPS, traditional paddy rice production system; GCRPS_sat_ and GCRPS_80%_, ground cover rice production system keeping root zone average soil water content near saturation and 80–100% of field water capacity, respectively. Horizontal error bars represent standard errors of 3 replications, and DAT is days after transplanting.

**Fig 7:**
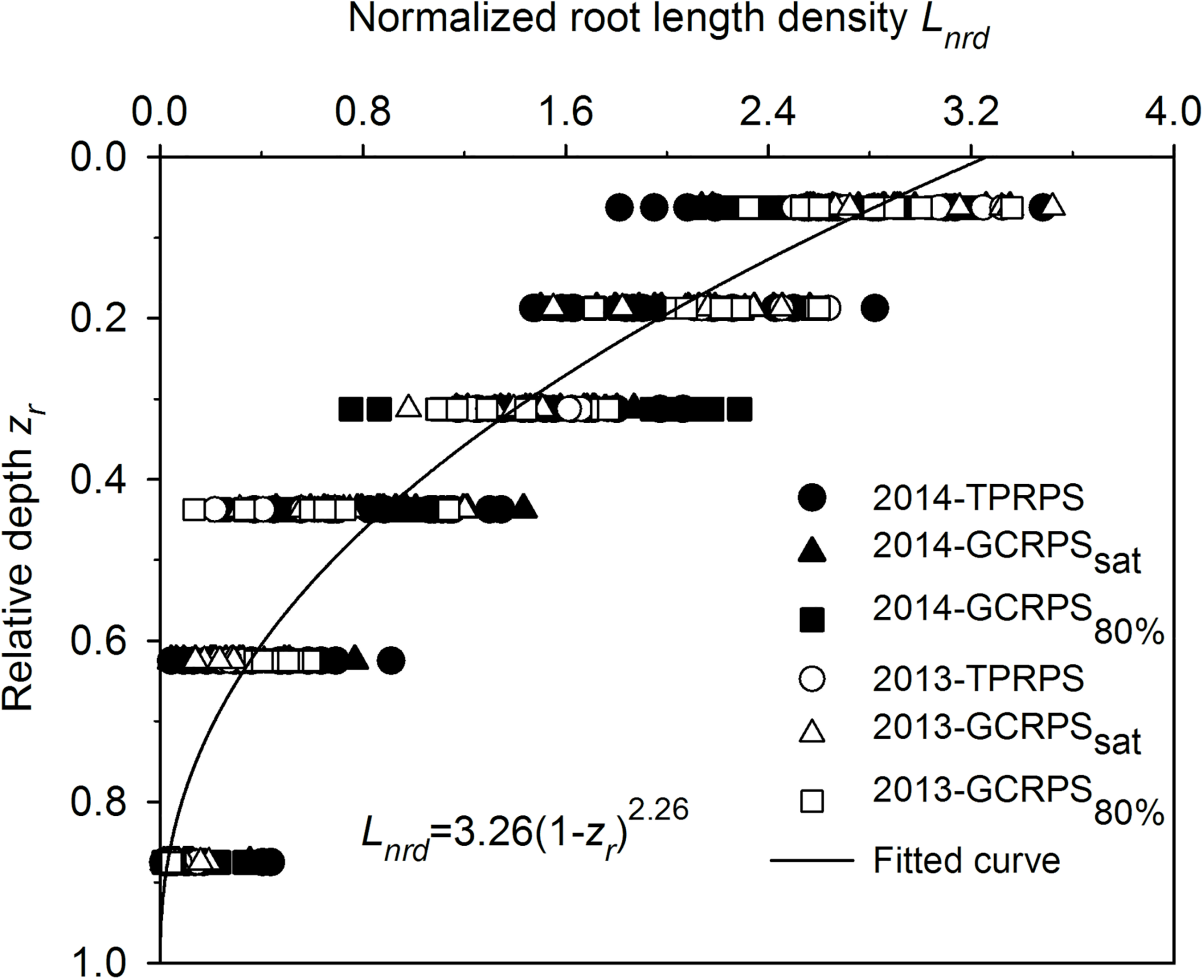
Measured experimental data and fitted general function regarding normalized root length density distributions of rice in 2013 and 2014 for Exp. 1. TPRPS, traditional paddy rice production system; GCRPS_sat_ and GCRPS_80%_, ground cover rice production system keeping root zone average soil water content near saturation and 80–100% of field water capacity, respectively.

In Exp. 2, since root distribution was restricted by the soil columns, root length density distribution was not considered. During the complete growing season in Exp. 1, root length density generally decreased with soil depth, and no significant difference was found in the 15–40 cm soil layer between the three treatments (Fig 6). Before mid-tillering, the root length density in the 0–15 cm soil layer was 109% greater under GCRPS in comparison to TPRPS. During the subsequent stages, the situation changed and the root length density under TPRPS and GCRPS_sat_ was significantly higher than that under GCRPS_80%_. The normalized root length density of rice, calculated by fitting the unknown parameter *a* in Eq (2) as 3.26 (*R*^*2*^ = 0.91, RMSE = 0.30) for all the normalized root length density data in both 2013 (with 126 data points) and 2014 (270 data points), decreased with depth (Fig 7).

**Fig 8:**
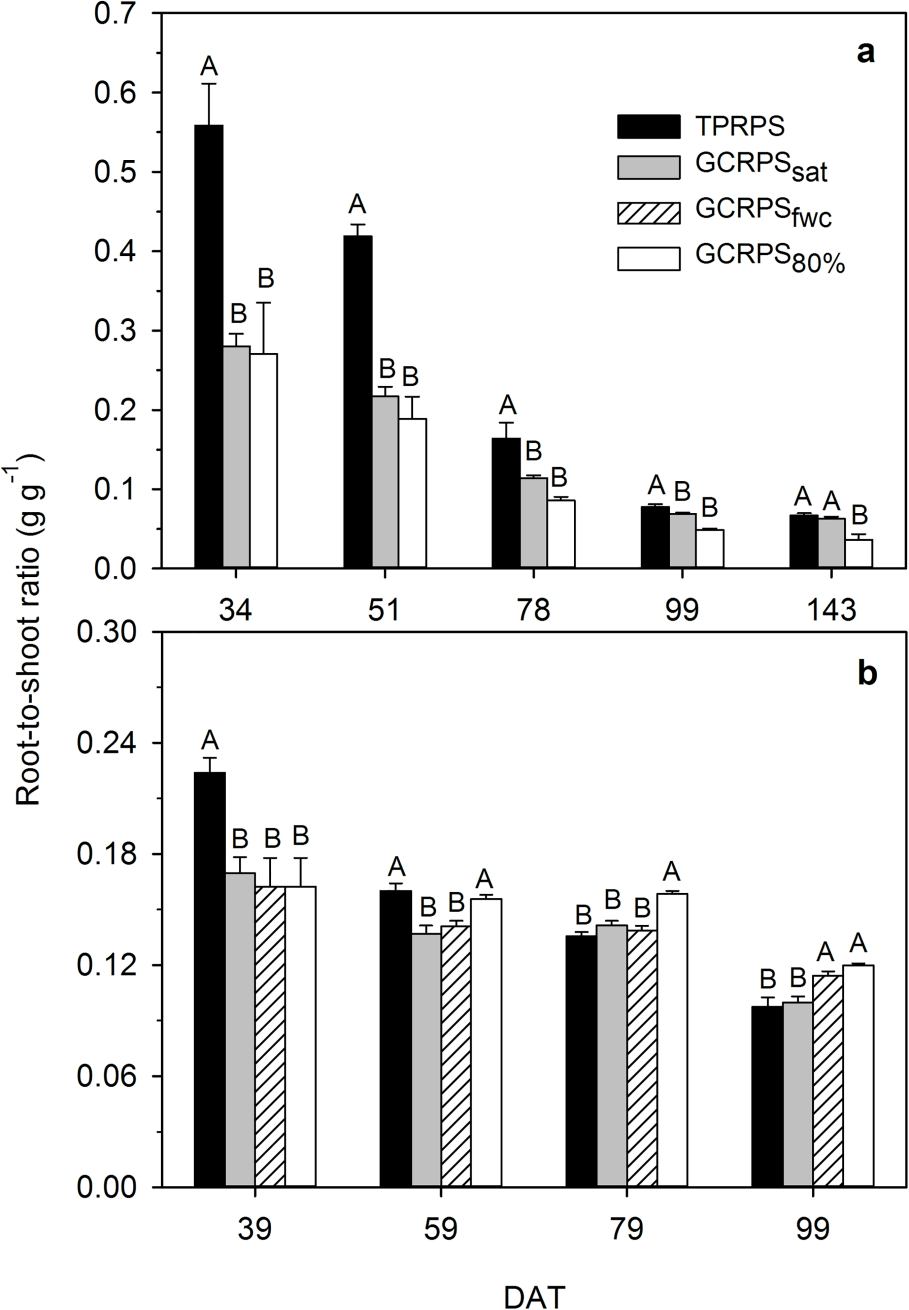
**Dynamics of root-to-shoot ratio for rice in Exp. 1 in 2014 (a) and Exp. 2 (b).** TPRPS, traditional paddy rice production system; GCRPS_sat_, GCRPS_fwc_ and GCRPS_80%_, ground cover rice production system keeping root zone average soil water content near saturation,100–120% and 80–100% of field water capacity, respectively. Different capital letters between treatments indicate significant difference at 0.05 probability level for given dates. Vertical error bars represent standard errors of 3 replications, and DAT is days after transplanting.

**Fig 9:**
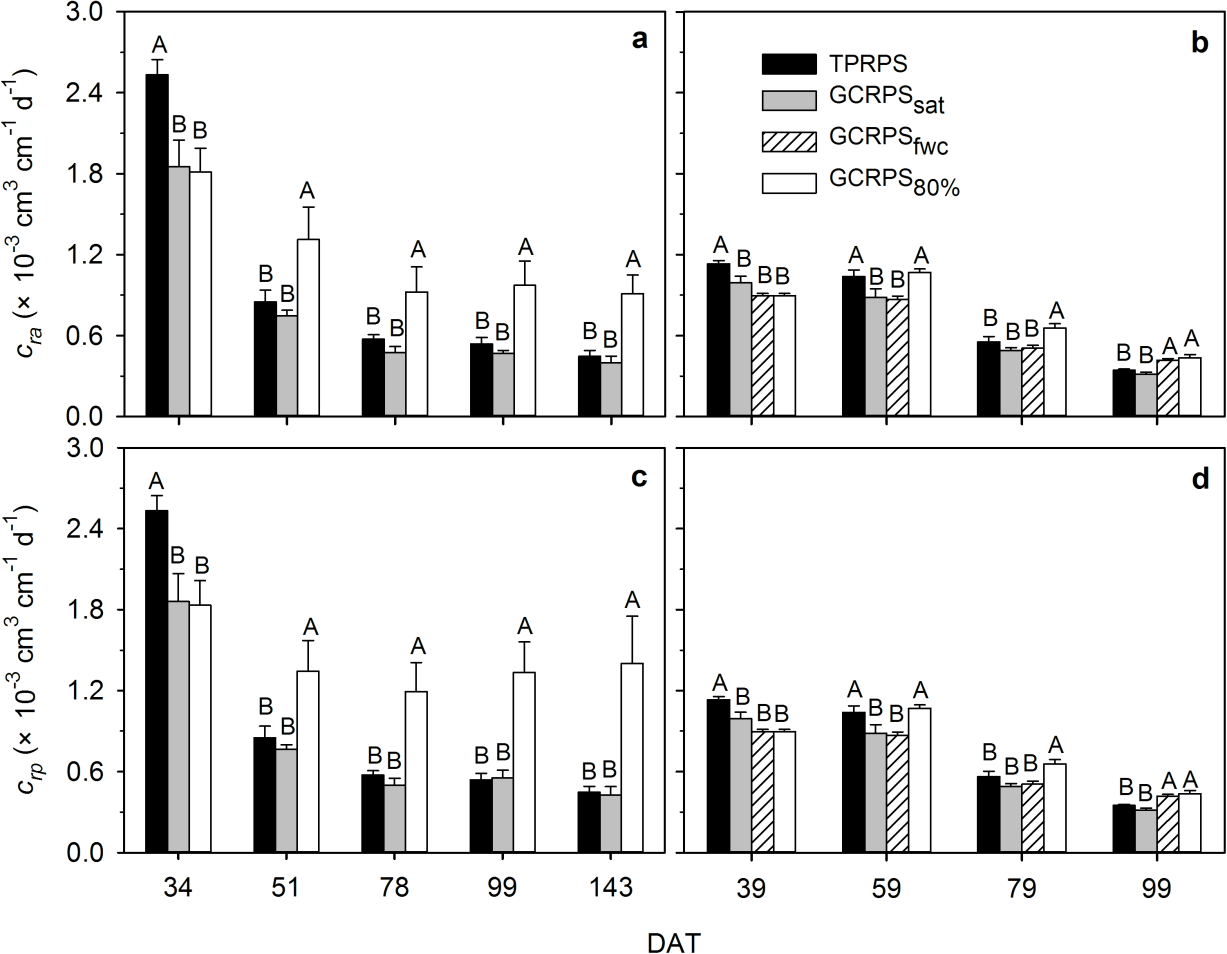
**Dynamics of actual (*c***_***ra***_**) and potential (*c***_***rp***_**) root water uptake coefficients for rice in Exp. 1 in 2014 (a and c) and Exp. 2 (b and d).** TPRPS, traditional paddy rice production system; GCRPS_sat_, GCRPS_fwc_ and GCRPS_80%_, ground cover rice production system keeping root zone average soil water content near saturation, 100–120% and 80–100% of field water capacity, respectively. Different capital letters between treatments indicate significant difference at 0.05 probability level at each date. Vertical error bars represent standard errors of 3 replications, and DAT is days after transplanting.

Under each treatment in the two experiments, root-to-shoot ratio and actual and potential root water uptake coefficients (*c*_*ra*_ and *c*_*rp*_) decreased generally with plant growth (Figs 8 and 9). The three parameters under TPRPS were significantly higher than those under GCRPS during the early growth period, and afterwards weakened gradually, becoming similar to GCRPS_sat_ and even finally lower than those under GCRPS_fwc_ and GCRPS_80%_ except the root-to-shoot ratios in Exp. 1.

## Discussion

### The effects of GCRPS on root growth, morphology and distribution

Root growth, morphology and distribution are closely related to many factors (genetics, growth stage, tillage, soil water and nutrients, soil temperature and other soil properties) [40]. Before mid-tillering, the saturated root zone under various treatments (Fig 1) indicated soil water was not the main limiting factor for rice root growth [19, 23]. The absolute soil temperatures themselves are of interest since in Exp. 1 they sometimes fell under 24°C which is considered unfavorable for normal root growth of rice [41]. The vigorous growth of the root system under GCRPS before mid-tillering (Fig 3A and 3B) therefore might be attributed to the significantly increased soil temperature [42]. After mid-tillering, when the warming effect under GCRPS weakened and even disappeared, soil water conditions became the predominant factor for root growth. In comparison to TPRPS, the weakened root growth after mid-tillering under GCRPS_sat_ might have been in response to mild water stress in the 0–20 cm soil layer (Fig 1A and 1B). Relative to TPRPS and GCRPS_sat_, the significantly limited root growth under GCRPS_80%_ and GCRPS_fwc_ and the earlier peaking of root dry weight at panicle initiation (Fig 3A and 3B) agreed well with those found by Zhang et al. [23] and are hypothesized to be driven by water stress [43–46].

Some previous studies have shown that stress from low temperatures might inhibit root lengthening and branching by slowing auxin transport in the root system [42]. Auxin has been proven critical for the initiation of lateral roots of *Arabidopsis thaliana* [47–49]. In Exp. 1, due to the higher soil temperature before mid-tillering, adventitious roots under GCRPS were 31% longer than those under TPRPS and lateral roots were even 101% longer (Fig 4A), leading to greater total root length (Fig 3C), smaller average diameter (Fig 3E) and higher specific root length (Fig 5A). The much less effect of the decreased average root diameter relative to that of the increased root length resulted in relatively larger root surface area under GCRPS (Fig 5C). After mid-tillering, soil moisture apparently replaced soil temperature as the main factor impacting root morphology. Previous studies reported a substantial increase of soil mechanical resistance with decreased soil water content, requiring plants to compensate by adjusting root anatomy (e.g. thickening cell walls, accelerating lignification of endodermis, exodermis and sclerenchyma) [50], and thus impeding root branching [51, 52]. Due to the lower soil water content under GCRPS (particularly GCRPS_80%_) compared to TPRPS, root lengthening slowed down dramatically (Fig 3X), especially that of lateral roots with 0–0.15 mm diameter (Fig 4B–4E), which resulted in a significant increase of average root diameter (Fig 3E) and a significant decrease of specific root length (Fig 5A). The much less effect of the increased average root diameter in comparison to that of the decreased root length led to decreased root surface area under GCRPS_80%_ (Fig 5C). The effects of GCRPS on root morphology (e.g. length, diameter, surface area, and specific root length), found in the field experiment (Exp. 1), were well testified by the greenhouse experiment (Exp. 2: Figs 3D and 3F, 4F–4I, 5B and 5D) and a pot experiment in Kato and Okami [45].

Although soil temperature and moisture conditions influenced root length density distribution significantly (Fig 6), all the normalized root length density distributions were similar and consistent, and could be described by a general function, regardless of water treatment, growth period or planting year (Fig 7). This finding is comparable to the statistical results for wheat [29, 53], and should be helpful for estimating root length density distribution and simulating soil water and nutrient transport in a soil-rice system. In this study, the calculated root dry weight per unit soil surface area or root length density under comparable TPRPS treatment was similar to that reported by Kato et al. [45] during corresponding growth periods, but it was much higher than some other cases where roots were rinsed by a hydropneumatic elutriation device with a sieve size bigger than 0.7 mm [54–56]. Differences might be due to different rice varieties, soil or climatic conditions, or the smaller sieve size (0.5 mm) used for manually washing the roots in Exps. 1 and 2.

Compared to TPRPS in Exp. 1, the lower root-to-shoot ratio under GCRPS before mid-tillering (Fig 8A) might result from the more significantly positive effect of the increased soil temperature on shoot [19] rather than root growth (Fig 3A). After mid-tillering, the root systems were of similar size under GCRPS_sat_ or even smaller under GCRPS_80%_ (Fig 3A), but the much larger canopies under GCRPS treatments [19] preserved the lower root-to-shoot ratios compared to TPRPS (Fig 8A). Similar effects of GCRPS on root-to-shoot ratio in Exp. 2 (Fig 8B) validated the results found in Exp. 1 except when shoot growth was greatly limited by serious drought stress under GCRPS_fwc_, most notably under GCRPS_80%_ [19]. A lower root-to-shoot ratio for rice under aerobic conditions has also been reported by previous studies [43, 57, 58], but is inconsistent with common perception. Usually, a higher root-to-shoot ratio is expected under drought stress for dry land crops with shoots limited more than roots, representing a mechanism to ensure root water uptake while restrict transpiration consumption [54, 59, 60]. The relatively lower root-to-shoot ratio found under GCRPS_80%_ however might be supported by a highly-efficient root system, which can take up enough water to meet plant needs even under moderate drought conditions.

### The effects of GCRPS on root water uptake capacity

In Exp. 1, since the positive effect of increased soil temperature on root branching and lengthening under GCRPS was much stronger than that on leaf growth and transpiration before mid-tillering, both the actual (*c*_*ra*_) and potential (*c*_*rp*_) root water uptake coefficients were significantly lower compared to those under TPRPS (Fig 9A and 9C). After mid-tillering, due to the lower soil water content under GCRPS_sat_ compared to TPRPS, the average actual transpiration rate was decreased 5.4% in spite of the larger leaf area of plants [19], while the average root length was 7.6% higher (Fig 3C), resulting in an average decrease of 13.2% in *c*_*ra*_ (Fig 9A). Under GCRPS_80%_, the average actual transpiration rate was decreased 8.8% after mid-tillering even as the leaf area was increased in comparison to TPRPS [19], but the average decrease of 44.7% in root length resulted in a significant increase in *c*_*ra*_ (Fig 9A). Relative to TPRPS and GCRPS_sat_, the enhancement of *c*_*rp*_ under GCRPS_80%_ was more remarkable than that of *c*_*ra*_ (Fig 9C) since the effect of drought stress was considered. The effects of GCRPS on *c*_*ra*_ and *c*_*rp*_, found in the field experiment, were corroborated in the greenhouse experiment (Fig 9B and 9D).

Root water uptake capacity is influenced by root morphological, anatomical, biochemical, molecular and genetic characteristics [40]. Plant roots with smaller diameter [61, 62] or under well-watered conditions [32, 63–65] are often expected to be more active in absorbing water and nutrients. Furthermore, Henry et al. [40] suggested that limiting root water uptake capacity might be a sensible choice for rice to improve water retention capacity under aerobic conditions. However, higher root hydraulic conductivity of rice under aerobic conditions has been reported repeatedly [66–68]. In particular, along with significantly smaller fine to coarse root ratio and specific root length, root water uptake capacity of rice under aerobic conditions was previously found to be 131% of that under TPRPS [45], which is comparable with the experimental findings in this study (Figs 3–5 and 9).

In addition to root water uptake capacity, root nutrient uptake capacity under a GCRPS might also be different from that under a TPRPS due to the changes of soil water and temperature conditions. The apparently complex mechanisms regarding the adjustment of root water or nutrient uptake capacity to environmental conditions are still incompletely understood. Recent research indicated that root uptake capacity of winter wheat was proportional to nitrogen mass in roots [32, 69, 70]. Additional study regarding the effects of GCRPS on rice root nitrogen content and mass, as well as its relationship with root uptake capacity, is expected to even further promote understanding the water-saving and yield-increasing mechanisms of this innovative technology.

## Conclusions

In comparison to TPRPS, enhanced surface soil temperature before mid-tillering and limited root zone soil water content at later growth stages led to significant adjustments to the root system under GCRPS. Before mid-tillering, roots under GCRPS grew and branched faster but water uptake rates per root length were limited. The subsequent situation was completely reversed, with root growth and branching limited while water uptake rates per root length improved under GCRPS, especially GCRPS_80%_. The adjustments in the root system allowed plants to allocate more photosynthetic products to shoots (e.g. lower root-to-shoot ratio) and ultimately to increase yield while saving water. However, further study is required to understand the physiological mechanism for this finding.

## Supporting information

S1 File The raw data for all figures and tables. (XLSX)
